# Agreement of tonometer for measuring intraocular pressure in Wistar rats: a systematic review

**DOI:** 10.1186/s40001-024-01927-z

**Published:** 2024-06-16

**Authors:** Anush Nayak, R. Deveswaran, S. Swati, L. Srividhya

**Affiliations:** 1 Optometry Division, Department of Allied Health Sciences, Faculty of Life and Allied Health Sciences, M S Ramaiah University of Applied Sciences, Bengaluru, India; 2 Department of Pharmaceuticals, Faculty of Pharmacy, M S Ramaiah University of Applied Sciences, Bengaluru, India; 3 Department of Biotechnology, Faculty of Life and Allied Health Sciences, M S Ramaiah University of Applied Sciences, Bengaluru, India

## Abstract

Glaucoma is the most common cause of irreversible blindness in the world. It is associated with elevated intraocular pressure (IOP). Fluctuations in tonometer readings have implications for glaucoma research, where accurate IOP measurements are vital for evaluating disease progression and treatment efficacy. Researchers should carefully select the appropriate tonometer and consider biases associated with different tonometers. Validation against standard measurements can improve IOP measurement accuracy in rat models. In conclusion, this systematic review will emphasize on the importance of selecting the appropriate tonometer for IOP measurement in rat models, considering potential biases and their implications for glaucoma research. Accurate and consistent IOP measurement in rat models is crucial for understanding glaucoma pathophysiology and developing effective treatments. This systematic review aims to assess agreement among tonometers used for measuring IOP in Wistar rat models primarily focusing on TonoLab, TonoVet, and Tono-pen. The review was conducted using PRISMA guidelines. Two articles were included for qualitative synthesis. The studies compared manometric IOP with TonoLab, rebound tonometer, and Tono-pen XL readings. It was observed that TonoLab consistently underestimated IOP, while Tono-pen XL tended to overestimate IOP compared to manometric measurements. The study’s findings will help researchers in making decisions about tonometer selection, leading to more reliable outcomes in glaucoma research using rat models. Further research, specifically RCT’s (randomized controlled trial) is needed to confirm the results and enhance IOP measurement precision in rat models.

**Supplementary Information:**

The online version contains supplementary material available at 10.1186/s40001-024-01927-z.

## Introduction

The incidence of irreversible blindness caused by glaucoma is increasing throughout the world and is associated with increase in intraocular pressure (IOP) [1–4]. To effectively manage glaucoma and its progression, it is essential to measure IOP objectively and consistently [5, 6]. Animal models, particularly rats, play a vital role in studying glaucoma’s cellular-level changes, especially in retinal ganglion cells and other retinal cell types [7, 8]. The ability to accurately and consistently measure IOP in animal models is crucial for understanding the pathophysiology of glaucoma and developing effective treatment options [1–3, 9]. Among various animal models, rats have been widely utilized to study glaucoma due to their anatomical and physiological ocular similarities to humans [4, 7, 8]. Still, the choice of a suitable tonometer for measuring IOP in rat models continues to be a critical concern [4, 10].

The scope of the review will encompass diverse tonometry methods, such as indentation, applanation, and rebound tonometry, which are commonly used in animal research. This includes the TonoLab, developed for mice and rats; the TonoVet, designed for dogs, cats and horses; and the Tono-pen, intended for use with all animal species [4]. Although these tonometers are widely used in studies involving rats and other animal models, their precise suitability for rat models remain unclear.

The primary goal of this systematic review is to shed light on the most accurate and reliable tonometer for measuring IOP in glaucoma-induced rat models. By enhancing the accuracy of IOP measurements in animal research, we can better understand the cellular-level changes associated with retinal ganglion cells and other retinal cell types, ultimately advancing our knowledge of glaucoma pathophysiology and facilitating the development of novel therapeutic approaches. This study’s findings will aid researchers in selecting the optimal tonometry method to establish a robust and reliable link between IOP and cellular-level changes in relation to the human retina.

## Methods

This systematic review follows the Preferred Reporting Items for Systematic Reviews and Meta-Analysis guidelines [12]. The protocol has been registered on the International Prospective Register of Systematic Reviews (CRD42023406666).

### Search strategy

Two independent reviewers (AN, LS) searched multiple databases with a week's gap between searches. We considered articles indexed in PubMed, Scopus, Cambridge Journals, Cumulative Index to Nursing and Allied Health Literature, Wiley Online Library, ProQuest, Taylor & Francis, Springer Link, Ovidmedline, SAGE Journals, Nature Journals and Web of Science for this systematic review. On May 17, 2023 last search was done. The search terms used were (“Glaucoma” OR “Hypertension” OR “Glaucoma rat model” OR “Ocular Hypertension*” OR “Angle Closure Glaucoma*” OR “Narrow-Angle Glaucoma”) AND (“Wister rat*” OR “Laboratory Rat*” OR “Wistar Rat*”) AND (“Reproducibility of Findings” OR “Reproducibility” OR “Reliability*” OR “Validity” OR “Agreement”) AND (“Ocular Tonometry” OR “Ocular Tension” OR “Tonometry”) Detailed information about the search strategy for PubMed keywords is provided in Appendix 1. The search was limited to articles written in English language.

### Study selection

Two reviewers independently (AN, LS) accessed the title and abstracts of the retrieved articles based on the predefined inclusion criteria. Articles with the main aim of assessing IOP in Wistar rats were included in the review. In addition, references were searched manually for yielded articles to include relevant articles in this current study. Rayyan was used for article screening to maintain blinding. The screening of articles was done using Rayyan to ensure the blinding in both phases. Comprehensive assessment of full-text review was performed independently by reviewers (AN, LS)- any disagreements were resolved through collaborative discussions involving authors (AN, LS, DR and SS). Articles were excluded based on the following criteria: (1) non-availability in English; (2) publication in books or gray literature; conference abstracts; and (3) studies deemed inadequate or of inappropriate quality.

### Data extraction

Two reviewers (AN, LS) independently extracted for (1) the general data: title of the study article, author names, year of publication; (2) methodology: study design, sample size, follow-up period; (3) type of glaucoma model; (4) devices to measure IOP; (5) range of IOP.

### Assessment of methodological quality and risk of bias

Critical appraisal tool “Systematic Review Centre for Laboratory Animal Experimentation (SYRCLE)” was used to assess the quality of the articles included in full-text review (Table S1) [11, 12].

## Results

**Fig. 1:**
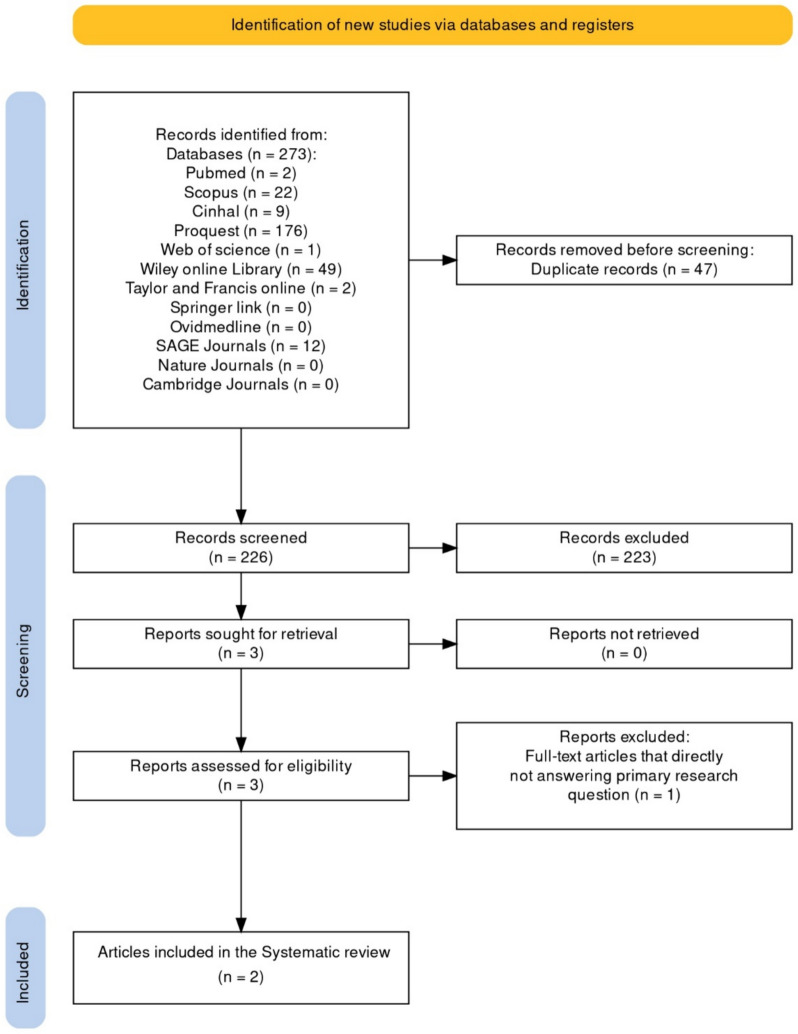
Details of articles search process. *SR* systematic review

A total of 273 titles and abstracts were identified from the search, after removing the duplicates, titles and abstracts of the retrieved articles were screened. Two full-text articles (Table S3) were included in the review according to the inclusion and exclusion criteria, details mentioned in Fig. 1.

**Fig. 2:**
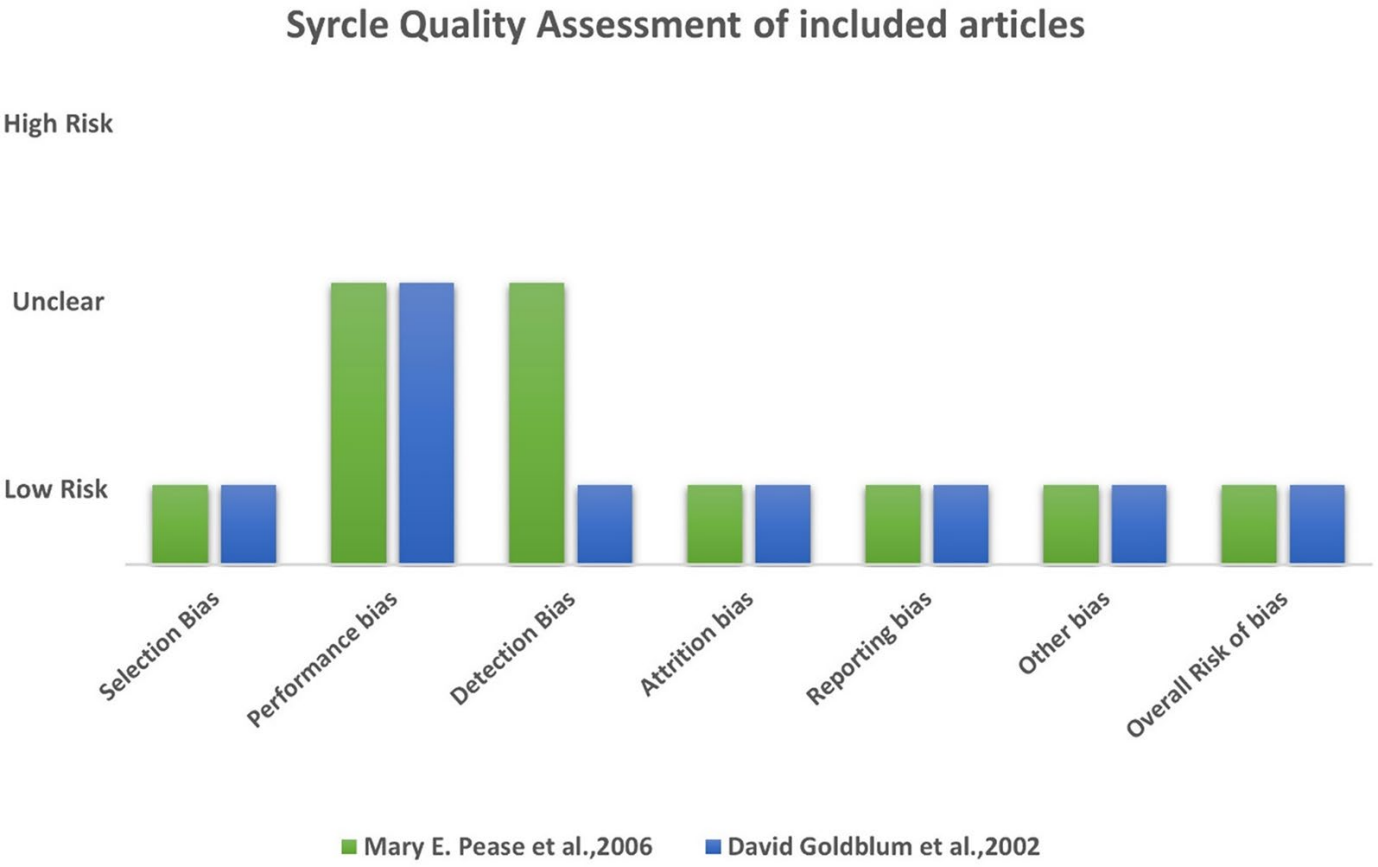
Methodological quality of included primary studies based on SYRCLE’s Risk of Bias tool

Included studies compared the reference standard tonometer, the manometer, with 3 different types of tonometers: TonoLab (Colonial Medical Supply, Franconia, NH); rebound tonometer (induction/impact (I/I) probe device); Tono-Pen XL (Mentor, Norwell, MA). Quality assessment results are summarized in Fig. 2. The overall risk of bias was determined to be low for both of these well-considered articles, instilling a sense of confidence in the robustness and reliability of their findings. Nonetheless, it is essential to acknowledge that, despite the overall positive stance in terms of risk of bias, a certain degree of ambiguity persists with regard to the assessment of performance bias.

Mary E. Pease et al. [13] compared the Tono-pen XL and TonoLab instruments in normal and glaucomatous rats. The objective was to evaluate the accuracy of these instruments in measuring IOP in rat eyes with known IOP levels determined using a manometer. The glaucoma model used in the study involved laser application to the trabecular meshwork. The researchers made three different comparisons during the study. Firstly, they compared the TonoLab and the manometer in 37 Wistar rat eyes over a follow-up period of 2–4 weeks, with the IOP ranging from 10 to 50 mmHg. The TonoLab was found to consistently underestimate IOP by 1 mmHg across the measured range. Secondly, the comparison was made between the Tono-pen XL and the Monometer in 26 Wistar rat eyes, with a follow-up period of 2–4 weeks and IOP ranging from 10 to 50 mmHg. The results showed that the Tono-pen XL tended to overestimate the manometric IOP by 1.5 mmHg at 10 mmHg and underestimate IOP at every level above 20 mmHg, with a 6 mmHg underestimate at 50 mmHg. Lastly, the researchers compared the TonoLab and the Tono-pen XL in 16 Wistar rat eyes, over a follow-up period of 2–4 weeks, with IOP ranging from 10 to 50 mmHg. In untreated eyes, they observed a mean difference of 3 mmHg, with the Tono-pen XL providing higher IOP readings (mean of 11 mmHg) compared to the TonoLab (mean of 8 mmHg). For glaucoma eyes, there were significant differences in the IOP measured by the two instruments. Specifically, the mean difference in paired readings on glaucoma eyes was greater when the Tono-pen XL was used first (5 mmHg) compared to when the TonoLab was used first (3.9 mmHg), although this difference was not statistically significant (*P* = 0.6).

In the second study conducted by David Goldblum et al. [14] investigated the accuracy and reproducibility of two tonometers, the I/I probe tonometer and the Tono-pen XL, in determining the true IOP in the eyes of living rats. The researchers compared the measurements obtained from these tonometers with the readings obtained from a manometric (true) IOP measurement. The study involved 18 Wistar rats, and the IOP range tested was between 8.9 mmHg and 20.2 mmHg. Three different comparisons were made: Rebound tonometer versus manometer, Tono-pen XL versus manometer, and I/I probe tonometer versus manometer. The results showed that both the Rebound tonometer and the Tono-pen XL tended to overestimate the manometric IOP values. Specifically, the Rebound tonometer had an average overestimation of 2.4 mmHg, with a range from + 7.2 mmHg to − 1.4 mmHg. On the other hand, the Tono-pen XL had an average overestimation of 3.6 mmHg, with a range from + 9.8 mmHg to − 3.2 mmHg. This study demonstrated that the Rebound tonometer and the Tono-pen XL showed some degree of overestimation compared to the true manometric IOP values when used to measure IOP in living rat eyes.

In these two studies comparing tonometers for measuring IOP in rat eyes, there were significant differences in the readings obtained from different tonometers and their accuracy in relation to manometric IOP measurements. In the study by Mary E. Pease et al., three tonometer (TonoLab, Tono-pen XL, and manometer) were compared in different combinations. The TonoLab was found to underestimate IOP by 1 mmHg across the measured range when compared to the manometer. On the other hand, the Tono-pen XL was found to overestimate manometric IOP by 1.5 mmHg at 10 mmHg, and it underestimated IOP at every level above 20 mmHg, with a 6 mmHg underestimate at 50 mmHg. When comparing TonoLab and Tono-pen XL, the Tono-pen XL gave higher IOP readings than TonoLab in untreated eyes with a mean difference of 3 mmHg. However, in glaucoma eyes, the difference in paired readings between the two instruments was not significant (5 mmHg when Tono-pen was used first and 3.9 mmHg when TonoLab was used first). In the study by David Goldblum et al., two tonometers (Rebound tonometer and Tono-pen XL) were compared against the manometer in Wistar rats. The Rebound tonometer overestimated the manometer by 2.4 mmHg (+ 7.2 mmHg to − 1.4 mmHg), while the Tono-pen XL overestimated the manometer by 3.6 mmHg (+ 9.8 mmHg to − 3.2 mmHg) across the range of IOP measured (8.9 mmHg to 20.2 mmHg).

Overall, both studies show that the accuracy of tonometers in measuring IOP in rat eyes varies, and the choice of tonometer may lead to different results. The TonoLab tends to underestimate IOP, while the Tono-pen XL tends to overestimate IOP in comparison to the manometric measurements. It is essential for researchers to consider these variations in tonometer readings when conducting studies involving IOP measurements in rat models, especially in the context of glaucoma research where precise IOP measurements are crucial for evaluating the disease progression and treatment efficacy.

## Discussion

Both studies demonstrated variations in tonometer readings and their accuracy in measuring IOP in rat eyes. The TonoLab tended to underestimate IOP, while the Tono-pen XL tended to overestimate IOP compared to manometric measurements. Such discrepancies in tonometer readings may have implications for glaucoma research, where precise IOP measurements are crucial for evaluating disease progression and treatment efficacy.

The findings from this systematic review underscore the importance of selecting an appropriate tonometer for IOP measurement in rat models. Researchers must consider the potential biases associated with different tonometers and choose the one that best aligns with the specific objectives of their study. Moreover, careful consideration of the context of IOP measurement, such as untreated versus glaucoma eyes, is vital to obtain reliable and meaningful results.

It is worth noting that both studies included in the review were of high methodological quality, and the overall risk of bias was determined to be low, instilling confidence in the reliability of their findings. However, some ambiguity remains concerning the assessment of performance bias, indicating a need for further research to validate and confirm the results.

*Variations in tonometer readings and accuracy:* Both studies conducted in Wistar rat models demonstrated variations in tonometer readings and their accuracy in measuring IOP compared to the reference standard, the manometer.

For the TonoLab, it consistently underestimated IOP across the measured range in one study, and in the other study, it provided lower IOP readings compared to the manometer. This consistent underestimation could lead to a potential underestimation of the true IOP values in rat eyes. On the other hand, the Tono-pen XL tended to overestimate IOP at lower pressures and underestimate IOP at higher pressures. This overestimation and underestimation could lead to inaccuracies in IOP measurements, particularly at different IOP levels relevant to glaucoma research.

*Implications for glaucoma research:* The discrepancies in tonometer readings observed in the studies have important implications for glaucoma research. Glaucoma is a complex disease, and measurement of precise IOP are crucial for understanding, evaluating disease progression and efficacy of treatment interventions. Inaccurate measurement of IOP can lead to misinterpretation of study results and potentially affects the treatment decisions.

Researchers conducting glaucoma studies in rat models need to be aware of these variations in tonometer readings and should carefully consider the choice of tonometer based on their study objectives. Depending on the research question, some tonometers may be more suitable than others. For instance, if a study aims to investigate IOP variations in a glaucoma-induced rat model, using a tonometer that tends to underestimate or overestimate IOP could potentially mask the true IOP elevation and its impact on disease progression.

*Selecting the appropriate tonometer:* Selecting an accurate tonometer is crucial in obtaining accurate and reliable IOP measurements in rat models. Researchers should consider the specific characteristics of each tonometer and the potential biases associated with their use. Additionally, factors such as the rat model used (normal eyes versus glaucoma-induced eyes) and the IOP range of interest should be carefully considered.

It may also be prudent for researchers to validate the chosen tonometer’s readings against the manometer or other reference standards in their specific experimental setup. This validation process can help researchers understand the tonometer’s performance and any inherent biases in their experimental conditions.

*Need for more agreement, repeatability, and reproducibility studies:* This systematic review highlights the variations in tonometer readings and their impact on IOP measurement in rat eyes. However, to further strengthen the evidence and ensure robustness in the selection of tonometers, there is a need for more randomized controlled trial’s (RCT’s) on agreement, repeatability, and reproducibility studies comparing different tonometers in Wistar rat models. These additional studies can provide valuable insights into the consistency and reliability of various tonometers, helping researchers make informed decisions about which tonometer to use for their specific experiments.

*Consideration of other tonometers:* The discussion section should acknowledge that in addition to the Tonolab and Tono-pen XL, there are several other tonometers currently available, such as Tono-Pen Avia Vet, and Kowa HA-2 portable tonometers. While this review focused on TonoLab and Tono-pen XL, it is important to highlight that these other tonometers could also be potential devices for IOP measurement in rat models. Researchers should explore the comparability and performance of these tonometers in future studies to expand the understanding of their suitability for specific research objectives.

## Conclusion

In conclusion, the systematic review’s findings underscore the importance of carefully selecting the appropriate tonometer for IOP measurement in rat models, especially in glaucoma research. The observed variations in tonometer readings and accuracy can impact the reliability of study results and influence the understanding of glaucoma pathogenesis and treatment outcomes. Researchers should be cautious in interpreting their results and consider potential biases associated with different tonometers. Further studies and validations may be necessary to confirm the findings and improve the precision of IOP measurements in glaucoma research involving rat models.

### Supplementary Information

Supplementary Material 1.

## Data Availability

Not applicable.
