# Supplementary material for: Agreement of tonometer for measuring intraocular pressure in Wistar rats: a systematic review
Source: Eur J Med Res. 2024 Jun 16;29:332. doi: 10.1186/s40001-024-01927-z (PMC11181524; doi:10.1186/s40001-024-01927-z)
Supplement: Supplementary file 1 — Supplementary Material 1. [file 40001_2024_1927_MOESM1_ESM.docx]

**Supplementary File:**

**Appendix 1:** Search strategy

**Table S1:** Methodological quality of included primary studies based on SYRCLE’s Risk of Bias tool.

**Table S2:** Excluded studies after full text review

**Table S3:** Details of the studies included in the systematic review

| **Appendix:1- Pubmed Search strategy (searched on 17th May 2023)** | | | |
| --- | --- | --- | --- |
| No. | Search no | Query | Results |
| 1. | #S1 | Search ("Glaucoma"[All Fields] OR "Hypertension"[All Fields] OR "Glaucoma rat model"[All Fields] OR "ocular hypertension*"[All Fields] OR "angle closure glaucoma*"[All Fields] OR "Narrow-Angle Glaucoma"[All Fields]) AND ("wister rat*"[All Fields] OR "laboratory rat*"[All Fields] OR "wistar rat*"[All Fields]) | [8911](https://www.ncbi.nlm.nih.gov/pubmed/?cmd=HistorySearch&querykey=9) |
| 2. | #S2 | Search ("Reproducibility of Findings"[All Fields] OR "Reproducibility"[All Fields] OR "reliability*"[All Fields] OR "Validity"[All Fields] OR "Agreement"[All Fields]) AND ("Ocular Tonometry"[All Fields] OR "Ocular Tension"[All Fields] OR "Tonometry"[All Fields]) | 1660 |
| 3. | #S1 AND S2 | Search: ("Glaucoma"[All Fields] OR "Hypertension"[All Fields] OR "Glaucoma rat model"[All Fields] OR "ocular hypertension*"[All Fields] OR "angle closure glaucoma*"[All Fields] OR "Narrow-Angle Glaucoma"[All Fields]) AND ("wister rat*"[All Fields] OR "laboratory rat*"[All Fields] OR "wistar rat*"[All Fields]) AND ("Reproducibility of Findings"[All Fields] OR "Reproducibility"[All Fields] OR "reliability*"[All Fields] OR "Validity"[All Fields] OR "Agreement"[All Fields]) AND ("Ocular Tonometry"[All Fields] OR "Ocular Tension"[All Fields] OR "Tonometry"[All Fields]) | 2 |

| **Table 1: Methodological quality of included primary studies based on SYRCLE’s Risk of Bias tool** | | | | | | | | |
| --- | --- | --- | --- | --- | --- | --- | --- | --- |
| **SN** | **Author, Year of publication** | ***Domain 1: Selection Bias*** | ***Domain 2: Performance bias*** | ***Domain 3: Detection Bias*** | ***Domain 4: Attrition bias*** | ***Domain 5: Reporting bias*** | ***Domain 6: Other bias*** | ***Risk of bias*** |
| 1 | Mary E. Pease et al.,2006 | LOW | UNCLEAR | UNCLEAR | LOW | LOW | LOW | LOW |
| 2 | Wan-Heng Wang et al.,2005 | UNCLEAR | UNCLEAR | LOW | HIGH | UNCLEAR | LOW | HIGH |
| 3 | David Goldblum et al.,2002 | LOW | UNCLEAR | LOW | LOW | LOW | LOW | LOW |

| **Table 2: Excluded studies after full text review** | |
| --- | --- |
| **Selected for review** | **Reason for exclusion** |
| \|  \| \| --- \| \| Wang, W.H., Millar, J.C., Pang, I.H., Wax, M.B. and Clark, A.F., 2005. Noninvasive measurement of rodent intraocular pressure with a rebound tonometer. Investigative ophthalmology & visual science, 46(12), pp.4617-4621. \| \|  \|  \| | **Topic not directly relevant** |

| **Table 3: Details of the studies included in the systematic review** | | | | |
| --- | --- | --- | --- | --- |
| **SN** | **Author, Year of publication** | ***Aim of the study*** | ***Type of Glaucoma model*** | ***Tonometers used*** |
| 1 | Mary E. Pease et al.,2006 | To compare TonoPen and TonoLab instruments in normal and glaucomatous rats against each other and to evaluate their readings in eyes of rats with mano-  metrically determined IOP. | Laser to trabecular meshwork | Tonolab, Tonopen XL and Manometer |
| 2 | David Goldblum et al.,2002 | Compares the accuracy and reproducibility of the I/I probe tonometer and the TonoPen XL in determining the true (manometric) IOP in the living rat eye. | Nil | Rebound Tonometer, Tonopen XL and Manometer |
